# Single-cell exome sequencing reveals multiple subclones in metastatic colorectal carcinoma

**DOI:** 10.1186/s13073-021-00962-3

**Published:** 2021-09-10

**Authors:** Jie Tang, Kailing Tu, Keying Lu, Jiaxun Zhang, Kai Luo, Haoxuan Jin, Lei Wang, Lie Yang, Weiran Xiao, Qilin Zhang, Xiaoling Liu, Xin yi Ge, Guibo Li, Zongguang Zhou, Dan Xie

**Affiliations:** 1 National Frontier Center of Disease Molecular Network, State Key Laboratory of Biotherapy, West China Hospital, Sichuan University, No. 17, Section 3, Renmin South Road, Chengdu, 610041 Sichuan China; 2 BGI-Shenzhen, Shenzhen, 518083 China; 3 Department of Gastrointestinal Surgery, West China Hospital, Sichuan University, No. 37, Guoxue Lane, Wuhou District, Chengdu, 610041 Sichuan China

**Keywords:** Colorectal cancer, Single-cell DNA sequencing, Tumour metastasis

## Abstract

**Background:**

Colorectal cancer (CRC) is a major cancer type whose mechanism of metastasis remains elusive.

**Methods:**

In this study, we characterised the evolutionary pattern of metastatic CRC (mCRC) by analysing bulk and single-cell exome sequencing data of primary and metastatic tumours from 7 CRC patients with liver metastases. Here, 7 CRC patients were analysed by bulk whole-exome sequencing (WES); 4 of these were also analysed using single-cell sequencing.

**Results:**

Despite low genomic divergence between paired primary and metastatic cancers in the bulk data, single-cell WES (scWES) data revealed rare mutations and defined two separate cell populations, indicative of the diverse evolutionary trajectories between primary and metastatic tumour cells. We further identified 24 metastatic cell-specific-mutated genes and validated their functions in cell migration capacity.

**Conclusions:**

In summary, scWES revealed rare mutations that failed to be detected by bulk WES. These rare mutations better define the distinct genomic profiles of primary and metastatic tumour cell clones.

**Supplementary Information:**

The online version contains supplementary material available at 10.1186/s13073-021-00962-3.

## Background

Colorectal cancer (CRC) is the third most commonly diagnosed cancer [1] and the fourth leading cause of cancer-related death worldwide [2]. Distant metastasis occurs in approximately 20% of newly diagnosed CRC patients [3] and is one of the major causes of patient death and a poor prognosis. However, the mechanism of the metastatic cascade in metastatic CRCs (mCRCs) remains unclear. Previous studies have revealed a number of driver genes, including APC, TP53, NRAS, and KRAS [4], that exhibit high mutational frequency in matched primary and metastatic tumours [5] and are acquired early in CRC carcinogenesis [6]. Studies based on targeted gene panels have also shown high mutational consistency between primary and metastatic CRC tumours [5, 7]. However, recent studies have shown high intra-tumoural heterogeneity (ITH) and subclone mixing in CRCs [6, 8], suggesting more complex dynamics in the cell population during metastasis. Therefore, previous strategies that focussed on sequencing bulk tissues present with considerable limitations.

Single-cell sequencing methods have emerged as powerful tools to resolve ITH and trace clonal lineages during tumourigenesis. With advances in experimental protocols and computational methods, it has become possible to identify genome-wide single-cell somatic mutation profiles [9, 10] and estimate the evolution of cell lineages [11–14]. Leung et al. reported a ‘late-dissemination model’ through single-cell lineage tracing from primary and liver mCRC samples based on a panel of 1000 cancer genes [10]. However, the limited number of genes in panel sequencing methods may restrict the evolutionary model of CRC metastasis.

To gain better insights into the divergence between primary and mCRC lesions, we generated bulk and single-cell whole-exome sequencing (scWES) data from primary tumours, metastatic tumours, and matched normal tissues of 7 patients with synchronous liver-limited mCRC. The comprehensive genomic analysis of mCRC provided important resources that allowed us to identify the subclones in each sample, and we uncovered a more detailed evolution model of mCRC.

## Methods

### Patients and clinical information

Seven patients with liver-limited mCRC were staged according to the American Joint Committee on Cancer version 7, initially diagnosed with CRC and received surgical treatment at West China Hospital of Sichuan University from October 27, 2015, to July 21, 2017. Information, including patient age, sex, ethnicity, pathology, and tumour stage, was collected from 7 patients (Table 1). Patient CRC5 received neoadjuvant chemotherapy before surgery, and all other patients did not receive any treatment before surgery. Primary and metastatic tumours and matched distal normal colon tissues were obtained during surgery. All samples were evaluated by two pathologists to determine the pathological diagnosis and tumour cellularity. Six of 7 patients were classified as having microsatellite-stable (MSS) CRC, and patient CRC5 was classified as having microsatellite-instability (MSI) CRC through immunohistochemistry (IHC). This study was approved by the Institutional Review Board of West China Hospital of Sichuan University, Chengdu, China (project identification code: 2017.114), and all patients provided written informed consent.

**Table 1:** Patient information covered in the article

Patient	Sex	Age	Grade	T	N	M	Stage	Primary tumour location	Site of metastasis
**CRC1**	Male	70	G3	2	2	1	IV	Left colon	Liver
**CRC2**	Male	64	G2	3	2	1	IV	Rectum	Liver
**CRC3**	Male	62	G2	2	0	1	IV	Left colon	Liver
**CRC4**	Female	69	G2	4	0	1	IV	Right colon	Liver
**CRC5**	Female	54	G2	3	0	1	IV	Left colon	Liver
**CRC6**	Male	54	G2	4	0	1	IV	Right colon	Liver
**CRC7**	Male	59	G2	3	0	1	IV	Left colon	Liver

### Tissue handling and tumour cell disaggregation

Resected tissues were transported in prechilled (4°C) RPMI-1640 medium to a clean bench as soon as possible and then rinsed with PBS several times. Clean tissues were minced into tiny pieces (<1 mm^3^) using scalpels, followed by transfer to 1 ml of a hypothermic protective solution (90% FBS and 10% DMSO). After procedural cooling in a −80°C freezer, the samples were stored in a −80°C freezer until use over the following several days. Frozen tissues in hypothermic protective solution were quickly thawed in a 37°C waterbath and subsequently subjected to centrifugation at 500 g for 5 min to remove superfluous hypothermic protective solution. Tissue pieces were digested in mixed digestion media composed of Hank’s solution, collagenase I (Gibco Cat. No. 17100-017) and collagenase IV (Gibco Cat. No. 17104-019) for 45–60 min, followed by mixing and inverting once every 5 min to dissociate cells thoroughly. Cell strainers (40 μm) were used to filter the digested cells. For each sample, a negative selection strategy was used to enrich cancer cells by cell sorting. Unwanted cells, such as leukocytes, vascular endothelial cells, and fibroblast cells, labelled with CD45, CD31, and PDGFRα, respectively, were filtered by flow cytometry. Finally, absolutely dissociated single active cells were selected under a microscope after flow cytometry sorting. These selected single cells in separate 0.2-ml tubes were amplified for downstream sequencing.

### Single-cell whole-genome amplification

All selected discrete single cells were subjected to whole-genome amplification using the REPLI-g Single Cell Kit (Qiagen, Cat. No. 150345). In brief, single cells were transferred to a 0.2-ml PCR tube with 4 μl of PBS; then, 3 μl of a denaturation solution mixture (1 M DTT, buffer DLB first mixed at a 1:11 ratio) was added and centrifuged briefly. Subsequently, the mixture was incubated for 10 min at 65°C was needed, and 3 μl of stop solution was added to end the reaction. A master mix containing 9 μl of H_2_O, 29 μl of REPLI-g sc Reaction Buffer, and 2 μl of REPLI-g sc DNA polymerase was then added to the above 10 μl of denatured DNA. Finally, the mixture was incubated at 30°C for 8 h and then at 65°C for 3 min. Amplified DNA was used for downstream sequencing.

### Bulk DNA extraction

Frozen specimens of proximal normal tissues, primary tumours, and liver metastases were separately subjected to bulk DNA extraction by using the DNeasy Blood & Tissue Kit according to the manufacturer’s manual (Qiagen). The extracted DNA was quantified on a Qubit Fluorometer and characterised by agarose gel electrophoresis. Qualified DNA was subsequently used for WES library construction.

### Whole-exome library construction and sequencing

Both tissues and single cells from varying locations in different patients were subjected to WES library construction. High-quality genomic DNA (single-cell whole-genome amplification products or bulk DNA from tissues) was used to construct the WES libraries.

In brief, the former procedures, including DNA fragmentation, size selection, end repair, A-tailing, adaptor ligation, and PCR amplification, were performed in a similar manner to that for WGS library construction. Subsequently, exome capture of the PCR results was performed with the BGI Exome Enrichment Kit v4.0 for enrichment. Pre-hybridization was performed at 95°C for 5 min, with a 65°C hold, followed by hybridization at 65°C for 24 h. After elution, 44 μl of the products was obtained. The post-PCR mixture included 100 μl of 2X KAPA HiFi HotStart Ready Mix, 6 μl of Ad-153-F (20 μM), and 6 μl of Ad-153-R (20 μM). Next, 44 μl of NF water was prepared and added to the above products. This 200-μl mixture was separated into two parts and amplified under the following programme: 95°C for 3 min; 13 cycles of 98°C for 20 s, 60°C for 15 s, and 72°C for 15 s; 72°C for 10 min; and 4°C overnight. After PCR, the products were purified using AMPure XP magnetic beads. The qualified WES libraries were then sequenced on a HiSeq-4000 system (Illumina) at an average coverage depth of ~300X for tissues and ~50X for single cells.

### WES data alignment and processing

All FASTQ files from the bulk and scWES data were subjected to adapter ligation and low-quality sequence trimming by using SOAPnuke version 1.5.6 [15] under the following parameters: ‘-l 5 -q 0.5 -n 0.1 -Q 2 -G --seqType 0’. We mapped clean reads to human reference build hg19 with Burrows-Wheeler Aligner (bwa) software version 0.7.12-r1039 [16]. Aligned reads were sorted and indexed using SAMtools version 1.2.1 [17], and the Genome Analysis Toolkit (GATK, version 4.0.10.1) [18] was used to realign reads to the genome and eliminate PCR duplicates.

### Single-nucleotide variant (SNV) and insertion/deletion (indel) calling in bulk samples

The final aligned bulk WES datasets were processed using the Genome Analysis Toolkit (GATK4) HaplotypeCaller [18] and Strelka2 [19] for germline genotyping. Specifically, for data processing, indel realignment, variant recalibration, and mapping quality of 40 were performed.

Both MutTect version 2 [20] and Strelka2 were used to call somatic mutations under default parameters, and only shared mutations detected by both software programmes were retained for subsequent analysis. To avoid potential low-quality SNV calling, mutations with low coverage (<10x) and those falling outside the exon regions were excluded. Variant annotation was performed using ANNOVAR [21] and CRAVAT 4.0 [22].

### Tumour purity

Tumour purity was calculated with PurBayes [23] using total reads and mutant allele supporting read counts for each somatic mutation from the tumour tissue WES data.

### SNV calling in scWES samples

For each patient, single-cell samples with target region coverage less than 75% or an average depth of 10× were excluded from the analysis. The GVCF model of GATK HaplotypeCaller was used to call SNVs in single-cell data. To generate highly confident SNV profiles from single-cell data, mutations were filtered and removed from the analysis by consensus filtering according to previous studies [10, 24]. In detail, SNV sites with low coverage (<10×) were labelled as missing values (NA). For SNV sites with 10–20× coverage, at least 10 variant reads were required. For SNV sites with 20–100× coverage, at least 30% of reads were required. For SNV sites with over 100× coverage, at least 20% of reads were required. Then, SNVs that occurred in fewer than 3 cells were located in clustered regions (more than one mutation detected within a 10-bp window) or were contained in germline and SNVs were removed from further analysis. For each SNV, the mutation frequency was defined as the percentage of cells that harboured the specific SNV.

### Phylogenetic tree

Mutational trees were calculated from single-cell mutation data using SiCloneFit [14] and redrawn using the ggtree R package [25]. The binary genotype matrix of single cells and point mutations with missing values was used for tree inference. SiCloneFit was run using a false-positive rate of 0.002 and an allelic dropout rate of 0.2.

### Cell lines

Five colon adenocarcinoma cell lines were purchased from American Type Culture Collection (ATCC; Manassas, VA, USA). All cell lines were cultured according to ATCC-recommended methods. Cells were maintained in a humidified incubator with 5% CO_2_ at 37°C. All cell lines were confirmed negative for mycoplasma by routine testing.

### RNA interference and cell migration assays

To silence genes related to metastasis, colon cancer cell lines were transfected with specific small interfering RNAs (siRNAs) or a scrambled sequence as a negative control (Transsheep Bio-Tech, Shanghai, China). All transfections were performed using the Lipofectamine RNAimax transfection reagent Lipofectamine 2000 (Thermo Fisher Scientific) according to the protocol of the manufacturer.

Migration assays were performed on CIM 16-well plates and with an xCELLigence RTCA-DP device (Acea Biosciences, Inc.). The complete medium was adjusted to the desired concentrations (final volume of 165 μl) and loaded into the lower chamber of the plate. Following cell attachment in the upper chamber, the upper wells were filled with 30 μl of opti-MEM medium, and the plate was incubated at 37°C for at least 1 h to pre-equilibrate and then used for a baseline measurement. Next, 100 μl of cell suspension was added to the upper wells. Cells were allowed to settle and adhere for 6 h in the incubator before they were transfected with 100 nM siRNA or scrambled RNA oligonucleotides. Six hours after transfection, the medium in the top wells was replaced with 100 μl of complete medium, and cell migration was continuously monitored over 24–48 h by measuring changes in electrical impedance. Cells were subjected to real-time monitoring every 15 min. Recording of the cell index (CI) as well as subsequent data analysis was performed using RTCA Software 1.2 (ACEA Biosciences Inc.).

## Results

### Patients with liver-limited mCRC

**Fig. 1:**
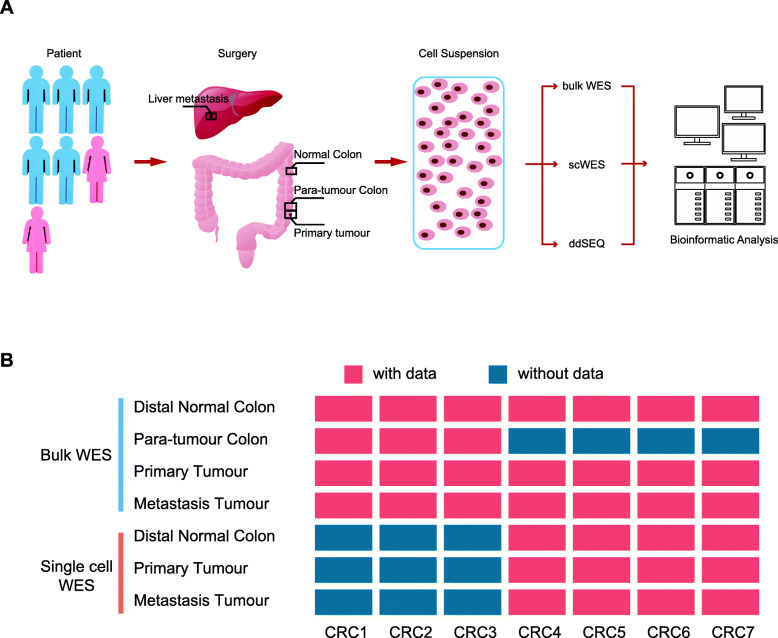
Design of the study. **A** Schematic diagram showing the experimental workflow of bulk and scWES data generation. **B** Heat map showing the sampling and data acquisition of this study. Red, with data; blue, without data

We collected tumour and para-tumour specimens from 7 patients with mCRC who had resectable liver metastasis. Six of the 7 patients were classified as having microsatellite-stable (MSS) CRC and had received no prior therapy, while one patient, CRC5, was classified as having microsatellite-instability (MSI) CRC and had received one course of neoadjuvant chemotherapy before surgery. Five of the 7 patients had no lymph node metastasis, indicating that the blood vessels were the preferred dissemination (Table 1). Tissues from the primary tumour, matched liver metastasis, para-tumour colon, and distal normal colon were processed to obtain a cell suspension (Methods, Fig. 1A). A total of 24 bulk WES and 321 scWES samples were sequenced on the Illumina platform, with high-quality data (Methods, Additional file 1: Table S1), which included single-cell and bulk sample pairs from four patients: CRC4, CRC5, CRC6, and CRC7 (Fig. 1B). However, the sample clustering analysis suggested that the bulk primary sample from CRC5 (CRC5_T) might have been mislabelled (Additional file 2: Fig S1A). Thus, we focussed on the 6 other patients from the bulk WES analysis.

### Patients showed various genetic alterations between matched bulk primary and liver metastatic lesions

**Fig. 2:**
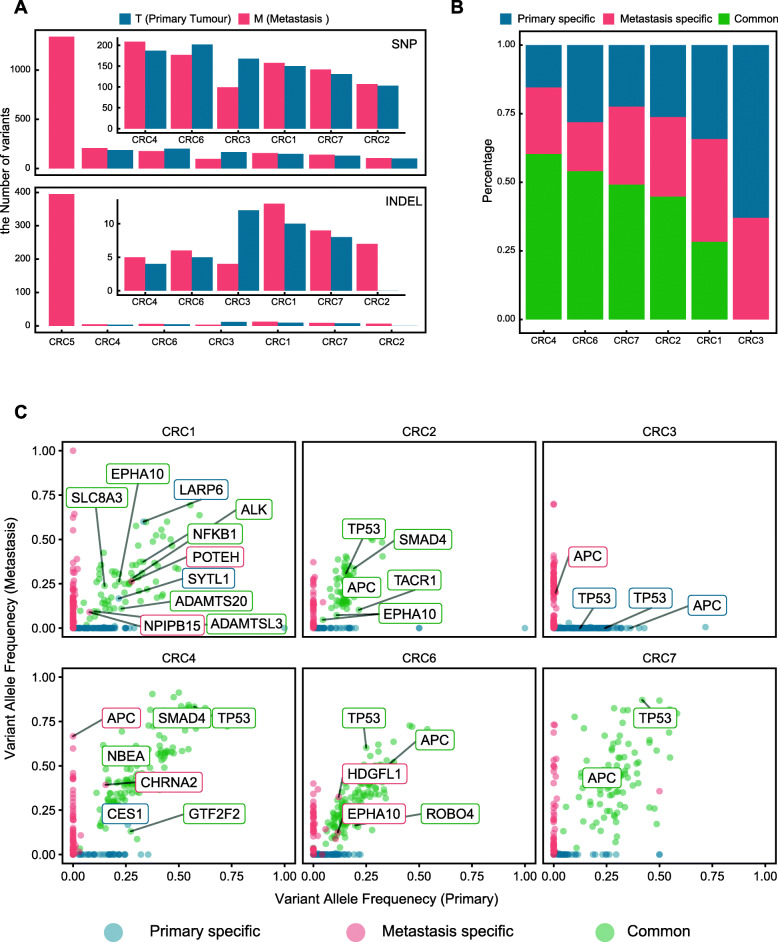
Overview of the bulk mutation profile comparison between matched primary and metastatic tumours. **A** Barplots show the number of SNVs (top) and indels (bottom) in the primary (blue) and metastatic (red) tumour tissues of 7 patients. **B** Barplot shows the percentage of primary tumour-specific (blue), metastatic tumour-specific (red), and common (green) SNVs. **C** Dot plots show the mutation allele frequencies of the SNVs in the primary and metastatic tumours

From the bulk sequencing data, we identified 2636 somatic SNVs (median, 153; range, 99 to 1335) and 456 somatic indels (median, 7; range, 0 to 395) in the 7 patients (Fig. 2A). A high concordance of the somatic mutation spectrum between primary and mCRC samples has been reported in previous studies [5, 10]. However, in our data, we observed a high concordance in the SNV spectrum (over 50% of common somatic SNVs) between the primary and metastatic tumours in only three patients: CRC4, CRC6, and CRC7. The other 3 patients (CRC2, CRC1, and CRC3) showed low concordance in the SNV spectrum between the two tumour lesions (Fig. 2B, Additional file 2: Fig S1B). Concerning common SNVs, bulk WES data reflected their different mutation allele frequencies between the primary and metastatic samples (Fig. 2C). Some important nonsynonymous SNVs, such as those in TP53, APC, and SMAD4, showed an increased variant allele frequency (VAF) in the metastatic samples (Fig. 2C). Our data also showed that the VAF of some primary or metastatic-specific SNVs in both tumour lesions was too low to be detected by SNV callers, such as SYTL1 and NPIPB15 in CRC1 (Fig. 2C).

Unexpectedly, patient CRC3, who presented with high somatic mutational divergence between the primary and metastatic lesions, was an exception. Almost no somatic mutations were detected in common between the primary and metastatic tumour lesions (Fig. 2B, C, Additional file 1: Table S2), although two tumour samples showed significant similarities in germline mutations (Additional file 2: Fig S1A). Because no other primary CRC disease was detected in CRC3, we speculate that the metastatic lesion of this patient may have been seeded by another recessive primary tumour that failed to be detected or had regressed. Alternatively, the inconsistency between the primary and metastatic tumour samples of other patients may be affected by tumour purity. Although we enriched cancer cells using flow cytometry sorting (Methods), we still found that some samples had low tumour purity based on the VAFs of somatic SNVs (Additional file 1: Table S3, Methods). To address these issues, we used scWES data in subsequent analyses.

### Single-cell WES data revealed rare mutations associated with tumour progression

While bulk sequencing data were advantageous in detecting somatic mutations in the dominant cell population, we usually failed to identify somatic mutations in rare cell populations. Therefore, we applied single-cell exome sequencing to 321 cells collected from the primary, liver metastasis, and proximal normal tissues of four patients (Methods, Additional file 1: Table S1; CRC4, CRC5, CRC6, and CRC7). The single-cell exome libraries were sequenced at high depth (median target depth, 81.72X; range, 34.67 to 137.87) and coverage (median target coverage, 90.6%; range, 39.14 to 98.38%) (Additional file 1: Table S1). To ensure the quality of the single-cell analysis, single-cell data with low coverage or depth were filtered (Methods), leaving 298 single cells for subsequent analysis. Notably, although we failed to analyse the bulk sample of CRC5 because it may have been mislabelled, all single-cell data of CRC5 showed high concordance between the normal and metastatic samples (Additional file 2: Fig S2A).

**Fig. 3:**
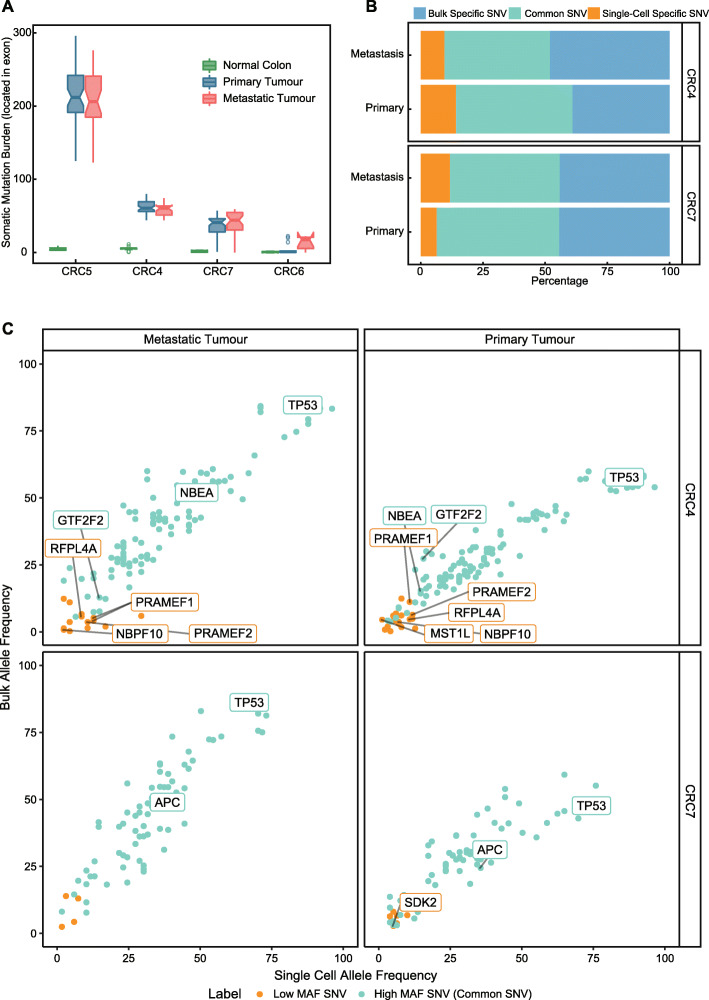
Overview of scWES data. **A** Boxplot shows the single-cell somatic SNV burdens of the primary tumour (blue), metastatic tumour (red), and normal colon tissue (green). **B** Barplots show the percentage of bulk-specific SNVs (blue), single-cell-specific SNVs (red), and common SNVs (green) between matched single-cell and bulk tumour samples of CRC4 and CRC7. **C** Dot plots show the correlation between SNV frequency in single cells (*x*-axis) and SNV mutation allele frequency in matched bulk samples (*y*-axis). The SNVs with low mutation allele frequencies defined in Additional file 2: Fig S2B are coloured blue (low-MAF SNVs). Common SNVs defined in both bulk and single cells are coloured green (common SNVs)

As expected, the cells from normal tissues presented with substantially fewer mutations (range, 1 to 14) than the cells from tumour tissues (median somatic mutation counts: CRC4, 84; CRC5, 284; CRC6, 2; and CRC7, 55, Fig. 3A). Consistent with previous research, the cells from MSI tumours (as in CRC5) had a significantly higher number of SNVs than those from MSS tumours (as in CRC4 and CRC7) (Fig. 3B, *p*<2.2e−16, Wilcoxon test). However, the total number of mutations in single cells between primary and metastatic tumours from CRC4, CRC7, and CRC5 did not show significant variation (Fig. 3A). Interestingly, we found very low somatic mutation burden in several single cells from the primary tumour lesion of CRC6 (Fig. 3A). These low-mutation burden cells may have originated from normal cells in the tumour tissue because they grouped with single cells from distal normal tissue after clustering (Additional file 2: Fig S2B). We next focussed on the single-cell mutation spectrum of CRC4 and CRC7 (with MSS tumours), as they had both single-cell and matched bulk data. In general, the single-cell data showed high concordance with the bulk data. Over 42.42% of somatic SNVs in CRC4 and CRC7 were shared between matched bulk and single-cell data (Fig. 3B, common SNVs, 42.42 to 49.29%, Additional file 1: Table S4). In single cells (Fig. 3B, single-cell-specific SNVs, 6.43 to 14.22%, Additional file 1: Table S4), we also identified 21.05% to 72.73% of single-cell-specific SNVs with a low frequency of reads with a matched mutation in the corresponding bulk data (Additional file 2: Fig S2B, low-MAF SNVs, Additional file 1: Table S4). Low-MAF SNVs were not identified in the bulk somatic SNV analysis because the somatic mutation calling algorithm could not confidently distinguish them from sequencing noise due to their low mutational frequency. As expected, both the common SNVs and the low-frequency SNVs showed high correlations between the mutational allele frequency in bulk data and the SNV percentage in the single-cell data (Methods, Fig. 3C). Interestingly, a number of low-MAF SNVs may be associated with cancer progression (Additional file 1: Table S4). For example, we identified low-frequency mutations in NBPF10, which was previously reported to be the most significant gene involved in breast cancer [26], in both the primary and metastatic tumours of CRC4 (Fig. 3C).

### Single-cell data revealed the cell population structures of primary and metastatic tumours

**Fig. 4:**
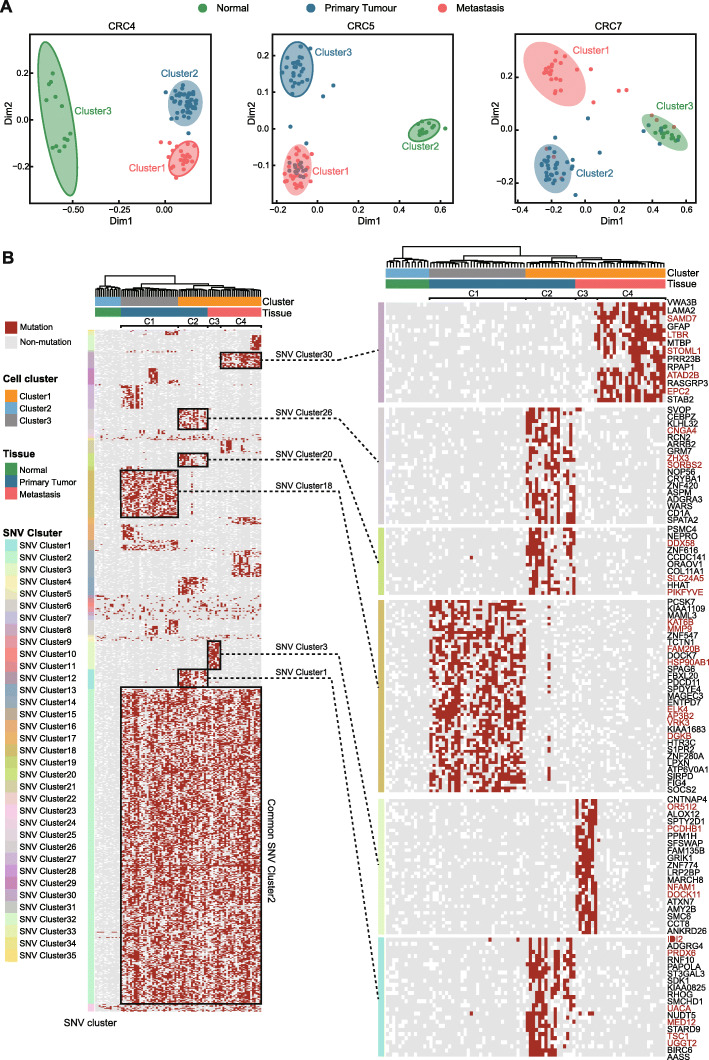
Single-cell mutation profile of matched primary and metastatic tumours. **A** Dot plots show multidimensional scaling analysis of the single-cell mutation profile, where each dot represents a single cell and is grouped by hierarchical clustering. Cells are colour-coded based on the sampling location. **B** Heatmap shows the single-cell somatic SNV profiles of CRC5. All single cells are in rows and sorted according to hierarchical clustering. SNVs are hierarchically clustered in columns. Red bars represent mutations, grey bars represent reference alleles, and white bars represent sites with a low sequencing depth (NA). Each SNV cluster is colour-coded as described in the legends. Genes with high-risk mutation (SIFT score < 0.05, POLYPHEN >0.85) were labelled in red

We then performed multidimensional scaling (MDS) analysis on the scWES data of patients CRC4, CRC5, and CRC7. Single cells from all patients were classified into 3 cell populations (Fig. 4A, Additional file 2: Fig S3A, B), corresponding to normal cells, primary tumour cells, and metastatic tumour cells. It is worth noting that we generated 90 high-quality single-cell datasets from the normal, primary, and metastatic tumours of CRC5, which was the patient with a hyper-mutation suffering from MSI disease. Both primary and metastatic cancer cells presented with a high mutation burden. According to the somatic SNVs, the tumour cells of CRC5 were classified into 4 major cell populations (Fig. 4B). Based on the appearance pattern of SNVs in these cancer cells, we identified 35 SNV clusters associated with 992 SNVs and 489 nonsynonymous gene mutations in total (Fig. 4B, Additional file 1: Table S5). Over 46.2% of SNVs (SNV cluster 2) commonly appeared in almost all tumour cells. DNA mismatch repair process-related genes, such as MLH1, and well-known CRC driver genes, such as PIK3CA [27], were included in the common gene set (Fig. 4B), suggesting their important functions in the generation of these cancer cells. Except for these common SNVs, the remaining SNVs (appearing in 3.95–39.47% of cancer cells) showed variant mutation patterns in different cancer cell clones. For example, two primary cancer cell clusters, ‘C1’ and ‘C2’, showed different mutation spectra. Primary cancer cells classified in C1 shared SNV clusters 18 and 15, including nonsynonymous mutations in transcription activator genes such as ELK4, KAT6B, and MAML3. These gene mutations were extremely rare in another primary CRC cluster, ‘C2’, which mainly harboured mutations in other SNV clusters, including nonsynonymous mutations in cell junction-related genes, such as DDX58, SDK1, and SORBS2, with significant functional impact scores based on SIFT (<0.05) [28] and POLYPHEN (p>0.85) [29] (Additional file 1: Table S5). Similar to primary tumour cells, the single tumour cells of metastases were also divided into two types: mCRC clusters C3 and C4 (Fig. 4B). Metastatic C3 cluster-specific mutations included nonsynonymous mutations in membrane proteins, such as PCDHB1 and AMY2B. Metastatic C4 cluster-specific mutations included those in SAMD7 and EPC2, with significant functional impacts. In addition to the main cell clones, we identified some small subclones located within primary CRC clones C1 and C4 with a small number of somatic mutations (Additional file 1: Table S5). Because patient CRC5 received one cycle of neoadjuvant chemotherapy, it was difficult to infer whether these tumour subclones were derived from the choice of chemotherapy drugs or from the tumourigenesis process. With the scWES data, it became easier to annotate tumour clones in each sample. These subclone-specific SNVs may be ignored or defined simply as somatic SNVs with low MAFs.

### Phylogenetic analysis of cell subclones showed the pattern of metastatic progression

**Fig. 5:**
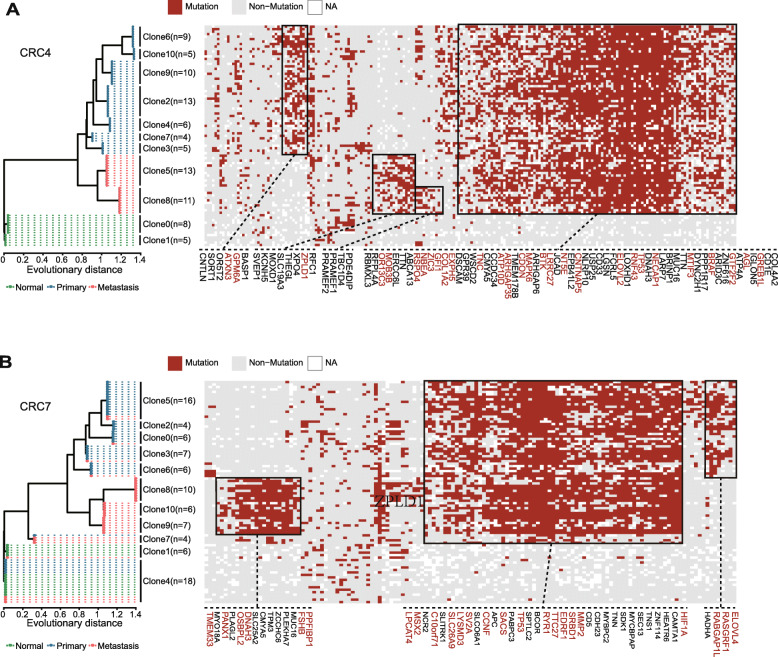
Cell lineage tree and subclone mutation profile of mCRCs. **A**, **B** Phylogenetic tree on the left panel shows single cells from CRC4 (**A**) and CRC7 (**B**). Cells are colour-coded based on the sampling location. The heatmap in the right panel shows the mutation profile of each cell in the phylogenetic tree. Gene mutations frequently occurring in primary and metastatic tumour cell clones are framed in blue and red, respectively. Mutations shared by most tumour cells are framed in black, and CRC-associated genes are labelled in red

We then studied the evolutionary process of mCRC at the single-cell level in patients CRC4 and CRC7. We constructed phylogenetic trees using SiClonefit (Methods) [14]. In patient CRC4, tumour cells were divided into 9 clones: 2 from the metastatic samples and 7 from the primary samples (Fig. 5A). Similar to the results we found in the subclone analysis of CRC5, all tumour cell clones shared a large number of SNVs, including those in some well-known CRC driver genes, such as TP53 and MAPK6 (Fig. 5A). These mutations may reflect the common origin of primary and metastatic tumour cells. To further explore the divergence of different tumour lineages, we identified cell clone-specific mutated genes for each tumour cell clone (Fig. 5A, Additional file 3: Table S6). These genes may be associated with the phenotype of primary or metastatic tumour clones. For example, we identified a high frequency of nonsynonymous mutations in ATXN3 in all primary tumour clones (clones 2, 3, 4, 6, 7, 9, and 10). This gene promotes breast cancer cell metastasis by deubiquitinating KLF4 [30], and its mutation in primary tumour cells may suppress metastasis. On the other hand, we identified nonsynonymous mutations that appeared specifically in the metastatic tumour cell clones (clones 5 and 8, Fig. 5A, Additional file 3: Table S6). These genes, such as RFC1, XPO4, THEGL, SLC19A3, TBC1D4, and KCNH5, may have specific functions in metastatic tumour cells.

Interestingly, in patient CRC7, all normal cells together with some primary and metastatic cells were classified into clones 1 and 4. Because these cells harboured only a small number of random SNVs (Fig. 5B), they may belong to normal cells, and the tumour source of cells in these clones may be derived from the normal infiltrating tissue in both primary and metastatic tumours. Similar to the mutation spectrum we observed in CRC4, a large percentage of SNVs were shared in all tumour cell clones, including SNVs on many well-known CRC driver genes, such as TP53 and APC (Fig. 5B). And we also observed two cell populations with different mutation profiles. In contrast to CRC4, in CRC7, the metastatic tumour cells appear in both cell populations. The first cell population is mainly composed of primary tumour cells, which formed 5 cell clones (clones 0, 2, 3, 5, 6). These clones harboured nonsynonymous mutations in genes that may be associated with tumour cell proliferation and invasion, such as RASGRF1 [31] and RABGAP1L [32] (Fig. 5B, Additional file 3: Table S6). The other cell population is composed of metastasis tumour cells only, which formed 3 tumour cell clones (clones 8, 9, and 10). These clones harboured nonsynonymous mutations in genes that may be associated with cell migration potential (Fig. 5B, Additional file 3: Table S6), such as PLEKHA7, which encodes an adherens junction protein [33] that plays an important role in modulating the dynamics of the tight junction barrier through the E-cadherin protein complex and microtubule-dependent mechanisms [34]. Gao et al. also reported that MUC16 has a higher frequency of nonsynonymous mutations in circulating tumour cells than in primary CRC cells [35].

### Metastatic tumour clone specific mutated genes exhibit high cell migration capacity

**Fig. 6:**
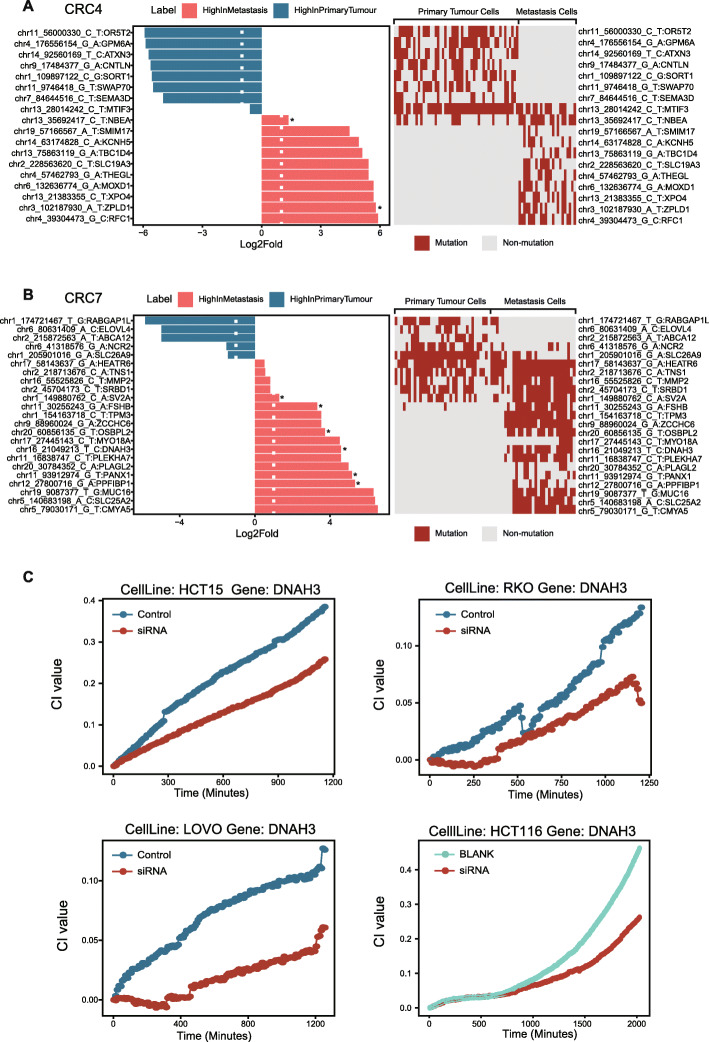
Mutation frequencies and functional validation of metastatic tumour cell-specific mutated genes. **A**, **B** Bar plots on the left panel show the comparison of mutated genes with significantly different mutation frequencies between metastatic tumour cell clones and primary tumour cell clones in CRC4 (**A**) and CRC7 (**B**). Fold change values are represented on the log2 scale. Primary and metastatic tumour cell clone-specific-mutated genes are colour-coded in blue and red, respectively. * represented mutation with SIFT <0.05, POLYPHEN >0.85. The heatmaps in the right panel show the gene mutation profiles of metastatic tumour cell clones and primary tumour cell clones from CRC4 (**A**) and CRC7 (**B**). **C** Real-time impedance traces for 4 CRC cell lines transfected with an siRNA against DNAH3 (colour-coded in red), the control siRNA (colour-coded in blue) and the blank group (colour-coded in green). The name of each cell line is labelled on the top of each panel

Notably, we observed large cell populations formed by metastatic tumour cells only in both newly diagnosed patients (CRC4 and CRC7). In total, we identified 24 genes with a high rate of nonsynonymous mutations in these metastatic tumour cell populations compared with the primary tumour cell populations (Fig. 6A, B, CRC4 10 genes, CRC7 14 genes, chi-squared test FDR<0.05, |log_2_-fold|>1), and 8 of these genes showed a high risk based on their functional impact scores (SIFT <0.05, POLYPHEN >0.85, Additional file 3: Table S6). As expected, most metastatic tumour-specific-mutated genes were rare in the primary tumour tissues of the Cancer Genome Atlas (TCGA) CRC cohort [4] (Additional file 2: Fig S4A). To further explore the functions of these genes, we performed a validation experiment in CRC cell lines with naturally occurring mutations according to the COSMIC database. There are 48 CRC cell lines in the COSMIC database that contain at least one mutation in a metastatic-specific mutated gene (Methods, Additional file 2: Fig S4B). We designed siRNAs to knock down the expression of genes with significant functional impact score (SIFT < 0.05 and POLYPHEN score > 0.85) or appear frequently only in the metastatic tumour clones of two patients. Then, we evaluated cell migration capacity via real-time cell analysis (RTCA) (Methods). In this way, if the gene of interest is related to cell migration, the migration ability of the cell should be changed after the target gene is knocked down. As expected, after knocking down DNAH3, the cell migration ability was reduced compared with the control samples in four COSMIC cell lines, including cell lines harbouring a naturally non-synonymous mutation (HCT-15 and HCT-116) and cell lines with a naturally synonymous mutation (Fig. 6C). Furthermore, knocking down CMYA5, PLEKHA7, and MYO18A also resulted in a decrease in cell migration capacity in more than one cell line (Additional file 2: Fig S5); we found similar cell phenotypes for TBC1D4 and SLC19A3, but in only one cell line (Additional file 2: Fig S5). According to our results, some highly frequently mutated genes may play an important role in the cell migration process, while other genes, such as MUC16, may influence the function of metastatic cancer cells in the other way, as its knockdown did not cause a significant change in cell migration (Additional file 2: Fig S5).

## Discussion

In this article, we generated WES data for mCRC tumours and normal tissues at both the bulk and single-cell levels. Our study provided insights into liver-limited mCRC; there are two critical points to note. First, the genomic profile could be more comprehensively detected through scWES than through bulk sequencing. With single-cell exome sequencing, we could identify rare mutations that were undetectable in the bulk data. Based on the single-cell data, we identified different subclones in both primary and metastatic tumour cells of the patient with MSI CRC, CRC5. Although single cells in each subclone shared a very large number of common mutations, a small number of subclonal-specific SNVs with low mutation frequencies in the whole cancer cell population could distinguish different cell clones. We also identified distinct cell populations in primary and metastatic tumour samples from CRC4 and CRC7. Again, the rare mutations that failed to be detected in bulk data because of their low allele frequencies acted as a remarkable source that defined the differences between the subclones in the primary and metastatic tumour cells in both patients.

We generated phylogenetic trees for the two patients with MSS CRC, CRC4, and CRC7. In both patients, tumour cells were divided into two major cell populations, which are mainly composed of tumour cells from primary and metastasis lesions, respectively. Most SNVs were shared among all of the tumour cells. Many of the common SNVs were found in genes associated with CRC progression, such as TP53 and APC. In addition to the common mutations, we observed some mutated genes appear specifically in primary tumour cell-enriched cell populations, such as those in AXIN3 and RASGRF1, were associated with tumour proliferation and invasion in previous studies. Interestingly, we also identified 24 nonsynonymous SNVs specific to metastatic cells. Metastatic cell-specific SNVs might have special functions in metastatic tumours. The knockdown of these genes in CRC cell lines, including DNAH3, TBC1D4, CMYA5, MYO18A, PLEKHA7, and SLC19A3, decreased cell migration capacity.

High genomic concordance between primary and mCRC tissues has been reported by previous research through gene panel sequencing [5, 10]. In this study, we observed common mutations between primary and mCRC tissues in most patients, except for CRC3, in whom the mutational profile was distinct between the primary and metastatic samples. This case may be explained by the ‘spatial heterogeneity of the primary tumour’, in which the common mutation was located in the primary tumour and was not sampled for sequencing.

This study was limited by the small number of patients (*n* = 7) and cells (*n* =321) and, therefore, was subject to potential sampling biases. We focussed on SNVs and small indels because their functionality can be predicted with multiple methods, and their heterogeneity has immediate clinical consequences for therapy selection. We withheld further analysis of copy number alterations at the single-cell level because of the random priming bias generated by MDA methods, as they are difficult to correct systematically [36].

## Conclusions

In summary, we generated bulk and scWES data from matched normal, primary, and metastatic tumour tissues of liver-limited mCRC patients. We identified rare mutations that were overlooked in bulk data using single-cell technology. These rare mutations are important in identifying different subclones in mCRC tissues. Only by sequencing a large number of genes at the single-cell level (i.e., by scWES) could these subclone-specific gene mutations be detected. In addition, we validated the functions of some metastatic subclone-specific-mutated genes in cell migration. However, the current study could be improved with a larger sample size and cell numbers.

## Supplementary Information

**Additional file 1: Supplementary tables S1~S5**

**Additional file 2: Supplementary figures**

**Additional file 3: Supplementary table S6**

**Additional file 4:.** Source data for Fig. 5

## Data Availability

Processed data, including genomic alterations and experimental validation, from CRC samples in this study have been deposited in the Genome Sequence Archive in BIG Data Center, Beijing Institute of Genomics, Chinese Academy of Science, under accession number HRA000762 (BioProject: PRJCA004804) that can be accessed at http://bigd.big.ac.cn/gsa-human [37]. Other high-level data can be accessed via the Data Centre of Precision Medicine in West China Hospital (https://pms.cd120.com/CRC/CRC_download.html) [38]. The source data underlying Fig. 5 is provided in Additional file 4. The raw bam files of patient CRC4, CRC6, and CRC7 are not provided due to patient privacy concerns.

## References

[1] Siegel RL, Miller KD, Jemal A (2018). Cancer statistics, 2018. CA Cancer J Clin.

[2] Bray F, Ferlay J, Soerjomataram I, Siegel RL, Torre LA, Jemal A (2018). Global cancer statistics 2018: GLOBOCAN estimates of incidence and mortality worldwide for 36 cancers in 185 countries. CA Cancer J Clin.

[3] Leufkens AM, van den Bosch MA, van Leeuwen MS, Siersema PD (2011). Diagnostic accuracy of computed tomography for colon cancer staging: a systematic review. Scand J Gastroenterol.

[4] Cancer Genome Atlas N (2012). Comprehensive molecular characterization of human colon and rectal cancer. Nature.

[5] Tan IB, Malik S, Ramnarayanan K, McPherson JR, Ho DL, Suzuki Y, Ng SB, Yan S, Lim KH, Koh D (2015). High-depth sequencing of over 750 genes supports linear progression of primary tumors and metastases in most patients with liver-limited metastatic colorectal cancer. Genome Biol.

[6] Hu Z, Ding J, Ma Z, Sun R, Seoane JA, Scott Shaffer J, Suarez CJ, Berghoff AS, Cremolini C, Falcone A (2019). Quantitative evidence for early metastatic seeding in colorectal cancer. Nat Genet.

[7] Brannon AR, Vakiani E, Sylvester BE, Scott SN, McDermott G, Shah RH, Kania K, Viale A, Oschwald DM, Vacic V (2014). Comparative sequencing analysis reveals high genomic concordance between matched primary and metastatic colorectal cancer lesions. Genome Biol.

[8] Sottoriva A, Kang H, Ma Z, Graham TA, Salomon MP, Zhao J, Marjoram P, Siegmund K, Press MF, Shibata D, Curtis C (2015). A Big Bang model of human colorectal tumor growth. Nat Genet.

[9] Xu X, Hou Y, Yin X, Bao L, Tang A, Song L, Li F, Tsang S, Wu K, Wu H (2012). Single-cell exome sequencing reveals single-nucleotide mutation characteristics of a kidney tumor. Cell.

[10] Leung ML, Davis A, Gao R, Casasent A, Wang Y, Sei E, Vilar E, Maru D, Kopetz S, Navin NE (2017). Single-cell DNA sequencing reveals a late-dissemination model in metastatic colorectal cancer. Genome Res.

[11] Jahn K, Kuipers J, Beerenwinkel N (2016). Tree inference for single-cell data. Genome Biol.

[12] Ross EM, Markowetz F (2016). OncoNEM: inferring tumor evolution from single-cell sequencing data. Genome Biol.

[13] Zafar H, Tzen A, Navin N, Chen K, Nakhleh L (2017). SiFit: inferring tumor trees from single-cell sequencing data under finite-sites models. Genome Biol.

[14] Zafar H, Navin N, Chen K, Nakhleh L (2019). SiCloneFit: Bayesian inference of population structure, genotype, and phylogeny of tumor clones from single-cell genome sequencing data. Genome Res.

[15] Chen Y, Chen Y, Shi C, Huang Z, Zhang Y, Li S, Li Y, Ye J, Yu C, Li Z (2018). SOAPnuke: a MapReduce acceleration-supported software for integrated quality control and preprocessing of high-throughput sequencing data. Gigascience.

[16] Li H, Durbin R (2009). Fast and accurate short read alignment with Burrows-Wheeler transform. Bioinformatics.

[17] Li H, Handsaker B, Wysoker A, Fennell T, Ruan J, Homer N, Marth G, Abecasis G, Durbin R (2009). Genome Project Data Processing S: The Sequence Alignment/Map format and SAMtools. Bioinformatics.

[18] McKenna A, Hanna M, Banks E, Sivachenko A, Cibulskis K, Kernytsky A, Garimella K, Altshuler D, Gabriel S, Daly M, DePristo MA (2010). The Genome Analysis Toolkit: a MapReduce framework for analyzing next-generation DNA sequencing data. Genome Res.

[19] Kim S, Scheffler K, Halpern AL, Bekritsky MA, Noh E, Kallberg M, Chen X, Kim Y, Beyter D, Krusche P, Saunders CT (2018). Strelka2: fast and accurate calling of germline and somatic variants. Nat Methods.

[20] Cibulskis K, Lawrence MS, Carter SL, Sivachenko A, Jaffe D, Sougnez C, Gabriel S, Meyerson M, Lander ES, Getz G (2013). Sensitive detection of somatic point mutations in impure and heterogeneous cancer samples. Nat Biotechnol.

[21] Wang K, Li M, Hakonarson H (2010). ANNOVAR: functional annotation of genetic variants from high-throughput sequencing data. Nucleic Acids Res.

[22] Masica DL, Douville C, Tokheim C, Bhattacharya R, Kim R, Moad K, Ryan MC, Karchin R (2017). CRAVAT 4: cancer-related analysis of variants toolkit. Cancer Res.

[23] Larson NB, Fridley BL (2013). PurBayes: estimating tumor cellularity and subclonality in next-generation sequencing data. Bioinformatics.

[24] Leung ML, Wang Y, Kim C, Gao R, Jiang J, Sei E, Navin NE (2016). Highly multiplexed targeted DNA sequencing from single nuclei. Nat Protoc.

[25] Yu G, Smith DK, Zhu H, Guan Y (2017). Lam TTY: ggtree: an R package for visualization and annotation of phylogenetic trees with their covariates and other associated data. Methods Ecol Evol.

[26] Hamdi Y, Boujemaa M, Ben Rekaya M, Ben Hamda C, Mighri N, El Benna H, Mejri N, Labidi S, Daoud N, Naouali C (2018). Family specific genetic predisposition to breast cancer: results from Tunisian whole exome sequenced breast cancer cases. J Transl Med.

[27] Huang D, Sun W, Zhou Y, Li P, Chen F, Chen H, Xia D, Xu E, Lai M, Wu Y, Zhang H (2018). Mutations of key driver genes in colorectal cancer progression and metastasis. Cancer Metastasis Rev.

[28] Ng PC, Henikoff S (2003). SIFT: predicting amino acid changes that affect protein function. Nucleic Acids Res.

[29] Adzhubei IA, Schmidt S, Peshkin L, Ramensky VE, Gerasimova A, Bork P, Kondrashov AS, Sunyaev SR (2010). A method and server for predicting damaging missense mutations. Nat Methods.

[30] Zou H, Chen H, Zhou Z, Wan Y, Liu Z (2019). ATXN3 promotes breast cancer metastasis by deubiquitinating KLF4. Cancer Lett.

[31] Chen H, Xu Z, Yang B, Zhou X, Kong H (2018). RASGRF1 hypermethylation, a putative biomarker of colorectal cancer. Ann Clin Lab Sci.

[32] Kawasaki N, Isogaya K, Dan S, Yamori T, Takano H, Yao R, Morishita Y, Taguchi L, Morikawa M, Heldin CH (2018). TUFT1 interacts with RABGAP1 and regulates mTORC1 signaling. Cell Discov.

[33] Pulimeno P, Bauer C, Stutz J, Citi S (2010). PLEKHA7 is an adherens junction protein with a tissue distribution and subcellular localization distinct from ZO-1 and E-cadherin. PLoS One.

[34] Paschoud S, Jond L, Guerrera D, Citi S (2014). PLEKHA7 modulates epithelial tight junction barrier function. Tissue Barriers.

[35] Gao Y, Ni X, Guo H, Su Z, Ba Y, Tong Z, Guo Z, Yao X, Chen X, Yin J (2017). Single-cell sequencing deciphers a convergent evolution of copy number alterations from primary to circulating tumor cells. Genome Res.

[36] Zhang CZ, Adalsteinsson VA, Francis J, Cornils H, Jung J, Maire C, Ligon KL, Meyerson M, Love JC (2015). Calibrating genomic and allelic coverage bias in single-cell sequencing. Nat Commun.

[37] Jie Tang, Kailing Tu, Dan Xie. Single-cell exome sequencing reveals multiple subclones in metastatic colorectal carcinoma. Genome Sequence Archive. https://bigd.big.ac.cn/gsa-human/browse/HRA000762. Accessed 24 May 2021. 10.1186/s13073-021-00962-3 PMC8434739 34507604

[38] Jie Tang, Kailing Tu, Dan Xie. Single-cell exome sequencing reveals multiple subclones in metastatic colorectal carcinoma. Data Centre of Precision Medicine in West China Hospital. https://pms.cd120.com/CRC/CRC_download.html. Accessed 26 Mar 2021. 10.1186/s13073-021-00962-3 PMC8434739 34507604

