# Supplementary material for: Single-cell exome sequencing reveals multiple subclones in metastatic colorectal carcinoma
Source: Genome Med. 2021 Sep 10;13:148. doi: 10.1186/s13073-021-00962-3 (PMC8434739; doi:10.1186/s13073-021-00962-3)
Supplement: Supplementary file 2 — Additional file 2: Supplementary figures [file 13073_2021_962_MOESM2_ESM.pdf]

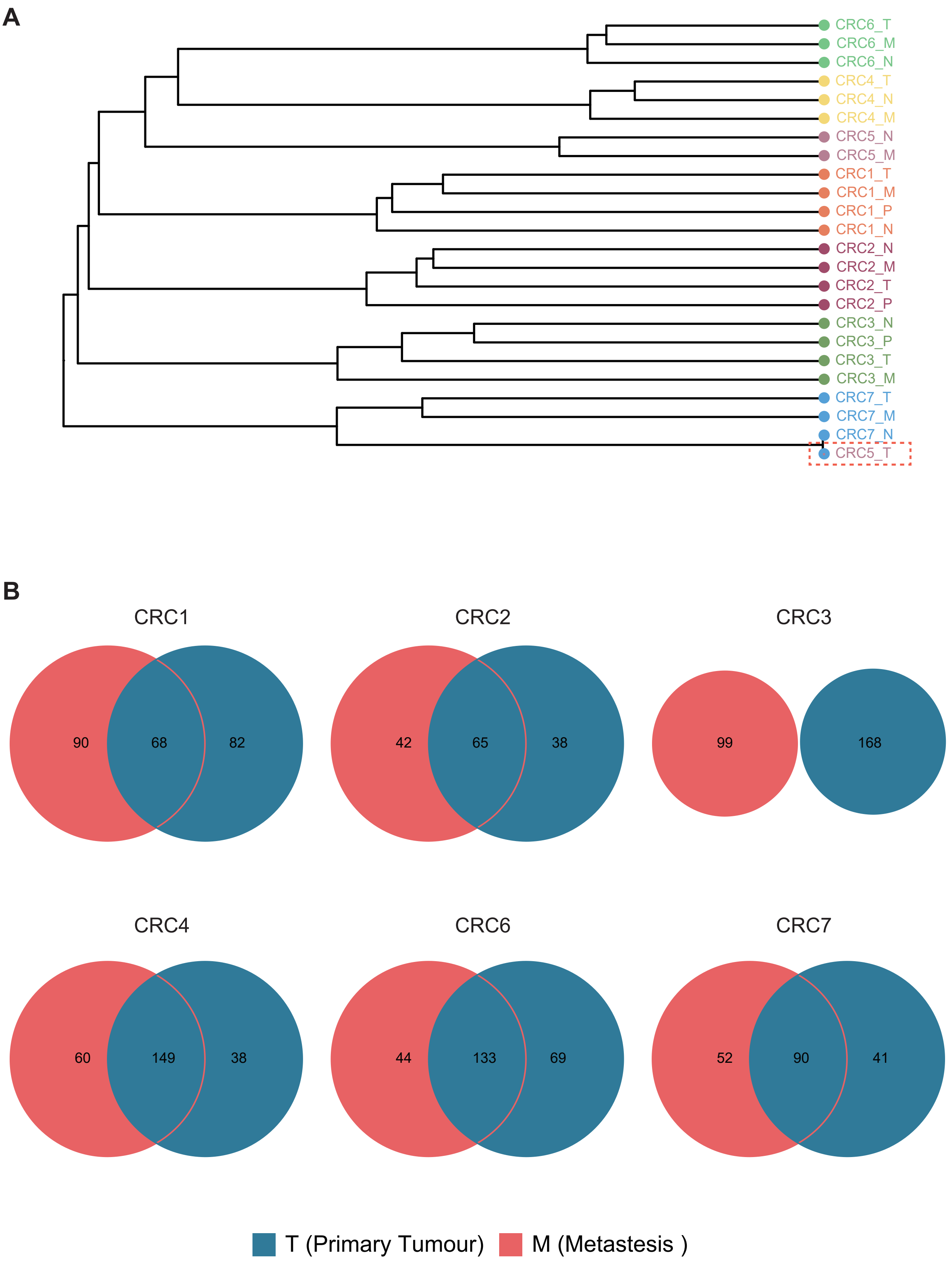

Fig S1: (A) Unsupervised hierarchical clustering trees of bulk WES samples based on SNVs detected by GATK. Different samples are colour-coded as described in the legends. **(B)** Venn diagrams reflect the total number of SNVs identified by exome sequencing of bulk tumour samples from the primary and metastatic tumours.

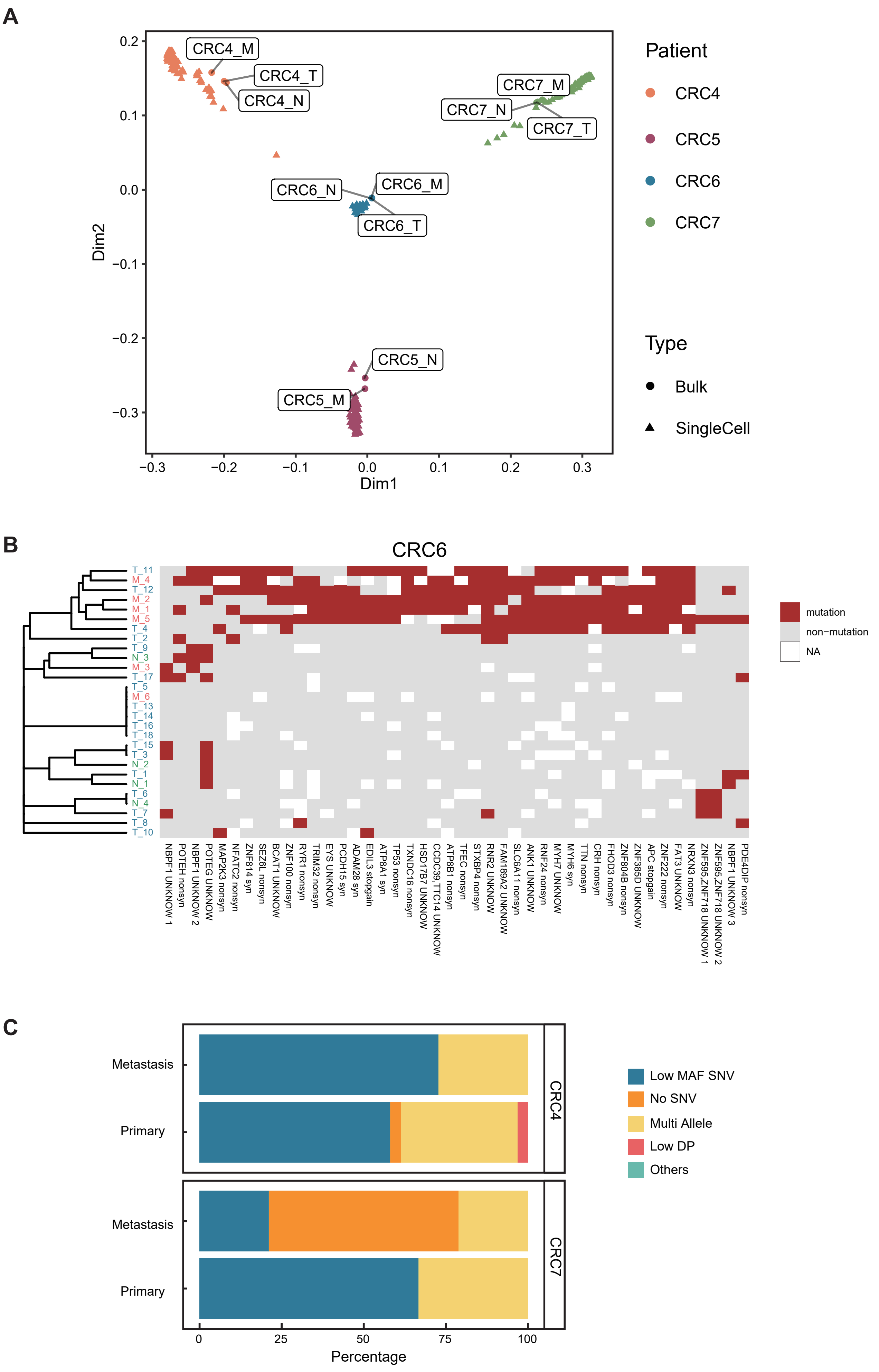
A
CRC4_M
CRC4_T
CRC4_N
CRC7_M
CRC7_N
CRC7_T
CRC6_N
CRC6_M
CRC6_T
CRC5_N
CRC5_M
Dim2
Dim1
Patient
CRC4
CRC5
CRC6
CRC7
Type
Bulk
SingleCell
B
CRC6
mutation
non−mutation
NA
NBPF1 UNKNOW 1
POTEH nonsyn
NBPF1 UNKNOW 2
POTEG UNKNOW
MAP2K3 nonsyn
NFATC2 nonsyn
ZNF814 syn
SEZ6L nonsyn
BCAT1 UNKNOW
ZNF100 nonsyn
RYR1 nonsyn
TRIM32 nonsyn
EYS UNKNOW
PCDH15 syn
ADAM28 syn
EDIL3 stopgain
ATP8A1 syn
TP53 nonsyn
TXNDC16 nonsyn
HSD17B7 UNKNOW
CCDC39,TTC14 UNKNOW
ATP8B1 nonsyn
TFEC nonsyn
STXBP4 nonsyn
RNR2 UNKNOW
FAM189A2 UNKNOW
SLC6A11 nonsyn
ANK1 UNKNOW
RNF24 nonsyn
MYH7 UNKNOW
MYH6 syn
TTN nonsyn
CRH nonsyn
FHOD3 nonsyn
ZNF804B nonsyn
ZNF385D UNKNOW
APC stopgain
ZNF222 nonsyn
FAT3 UNKNOW
NRXN3 nonsyn
ZNF595,ZNF718 UNKNOW 1
ZNF595,ZNF718 UNKNOW 2
NBPF1 UNKNOW 3
PDE4DIP nonsyn
C
Metastasis
Primary
Metastasis
Primary
CRC4
CRC7
Percentage
Low MAF SNV
No SNV
Multi Allele
Low DP
Others

Fig S2: (A) Dot plot shows multidimensional scaling analysis of bulk and single-cell samples from CRC4, CRC5, and CRC7. Bulk samples are shown in circles, and single-cell samples are shown in triangles. Patient IDs are colour-coded as described in the legends.**(B)** Heatmap shows the mutation spectrum of single cells from patient CRC6. All cells are in rows and sorted according to hierarchical clustering. “T” means primary tumour coming cells, “M” means metastasis tumour coming cells, and “N” means distal normal colon tissue coming cells. Rad bars represent mutations, grey bar represents reference alleles, and white bars represent sites with low sequencing depth (NA). **(C)** Bar plots show the different types of SNVs identified in the single-cell-specific SNVs of CRC4 and CRC7. Blue bars represent single-cell-specific SNVs with low mutation allele frequencies in bulk data (low-MAF SNVs), red bars represent SNVs with low sequencing depths in bulk samples (low DP), yellow bars represent SNVs with multiple alternative alleles in bulk data (multi allele), orange bars represent bulk undetectable SNVs (no SNV), and cyan bars represent other undetectable SNVs in bulk data because of other reasons (others).

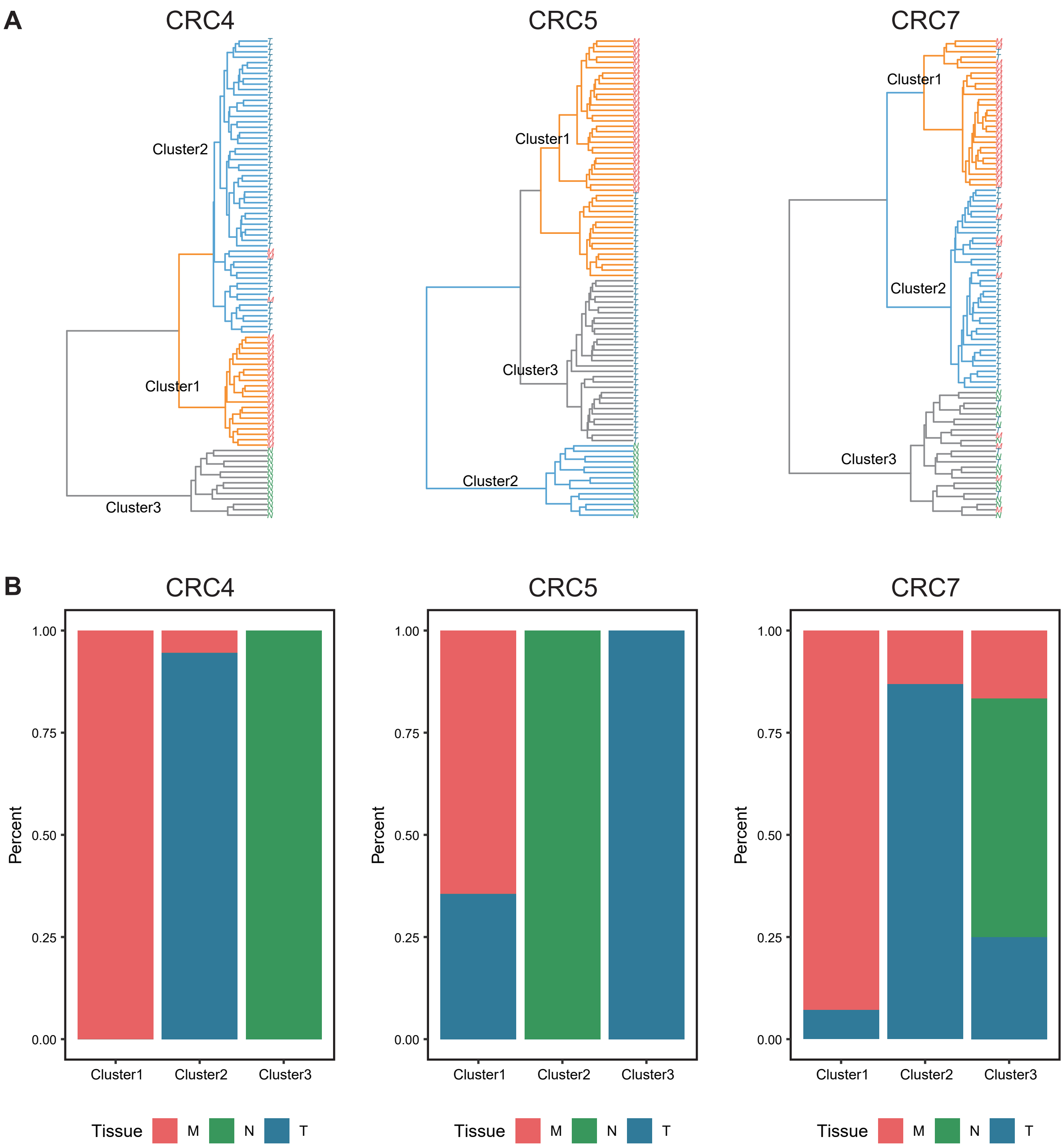

Fig S3: (A) Unsupervised hierarchical clustering trees of CRC4, CRC5, and CRC7. Each node representing a single cell is colour-coded based on the sampling location. Each branch representing a cell cluster is colour-coded based on Figure 4A. **(B)** Bar plots show the distribution of cells sampled from the normal colon tissues (green), primary tumours (blue), and metastatic tumours (red) of CRC4, CRC5, and CRC7.

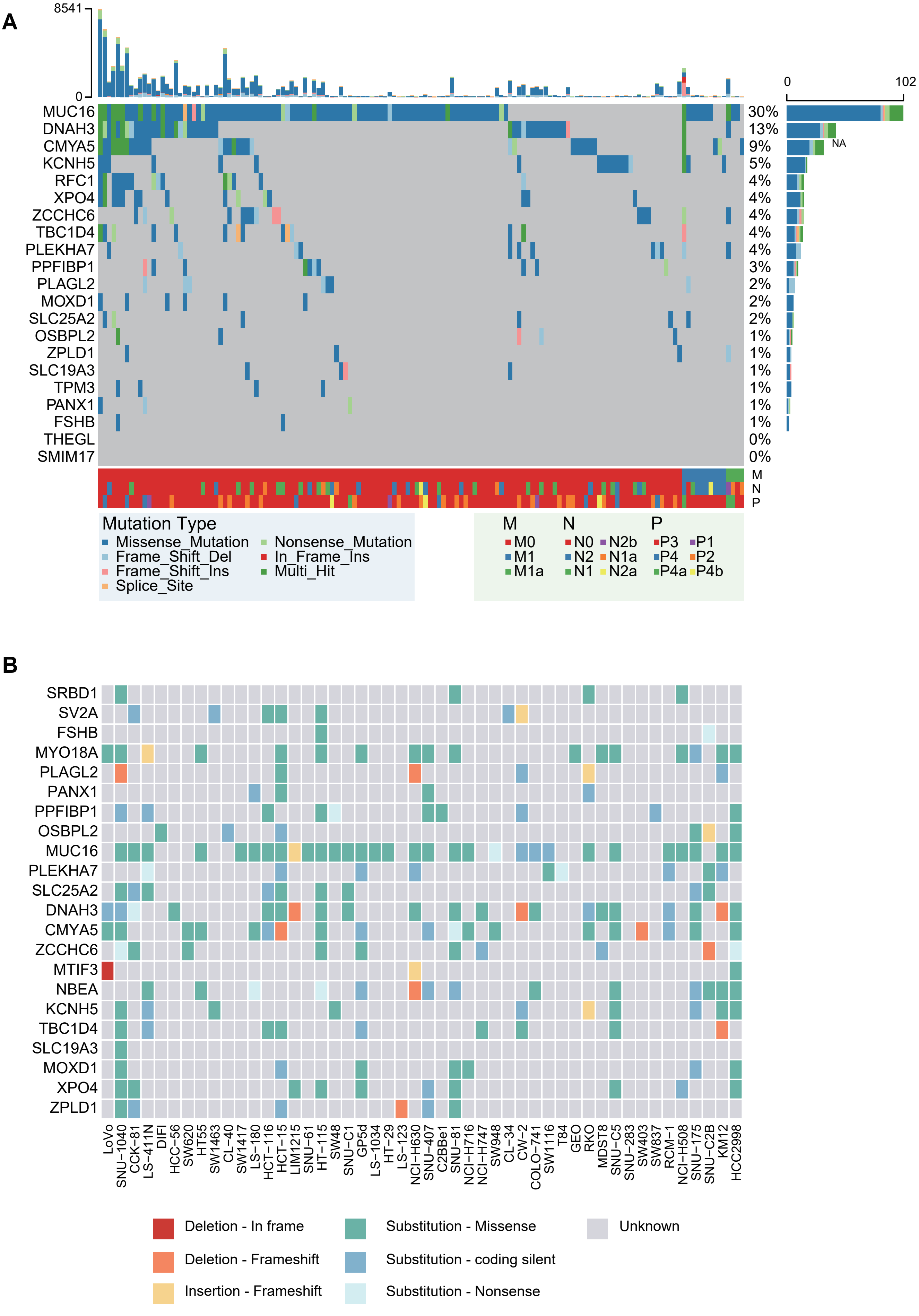
A
8541
0
102
NA
MUC16
DNAH3
CMYA5
KCNH5
RFC1
XPO4
ZCCHC6
TBC1D4
PLEKHA7
PPFIBP1
PLAGL2
MOXD1
SLC25A2
OSBPL2
ZPLD1
SLC19A3
TPM3
PANX1
FSHB
THEGL
SMIM17
30%
13%
9%
5%
4%
4%
4%
4%
4%
3%
2%
2%
2%
1%
1%
1%
1%
1%
1%
0%
0%
M
N
P
Mutation Type
Missense_Mutation
Frame_Shift_Del
Frame_Shift_Ins
Splice_Site
Nonsense_Mutation
In_Frame_Ins
Multi_Hit
M
M0
M1
M1a
N
N0
N2
N1
N2b
N1a
N2a
P
P3
P4
P4a
P1
P2
P4b
B
SRBD1
SV2A
FSHB
MYO18A
PLAGL2
PANX1
PPFIBP1
OSBPL2
MUC16
PLEKHA7
SLC25A2
DNAH3
CMYA5
ZCCHC6
MTIF3
NBEA
KCNH5
TBC1D4
SLC19A3
MOXD1
XPO4
ZPLD1
LoVo
SNU−1040
CCK−81
LS−411N
DIFI
HCC−56
SW620
HT55
SW1463
CL−40
SW1417
LS−180
HCT−116
HCT−15
LIM1215
SNU−61
HT−115
SW48
SNU−C1
GP5d
LS−1034
HT−29
LS−123
NCI−H630
SNU−407
C2BBe1
SNU−81
NCI−H716
NCI−H747
SW948
CL−34
CW−2
COLO−741
SW1116
T84
GEO
RKO
MDST8
SNU−C5
SNU−283
SW403
SW837
RCM−1
NCI−H508
SNU−175
SNU−C2B
KM12
HCC2998
Deletion - In frame
Deletion - Frameshift
Insertion - Frameshift
Substitution - Missense
Substitution - coding silent
Substitution - Nonsense
Unknown

Fig S4: (A) OncoPlot shows the distribution of metastatic tumour cell-specific mutated genes in the TCGA CRC cohort. The top panel shows individual tumour mutation rates, the middle panel shows metastatic tumour cell-specific mutated genes (chi-square test FDR<0.05), and the bottom panel details the TNM stage of 145 CRC patients. **(B)** Heatmap showing the gene mutation profiles of metastatic tumour clone-specific mutated genes in COSMIC CRC cell lines. Different types of gene mutations are colour-coded based on the colour bar.

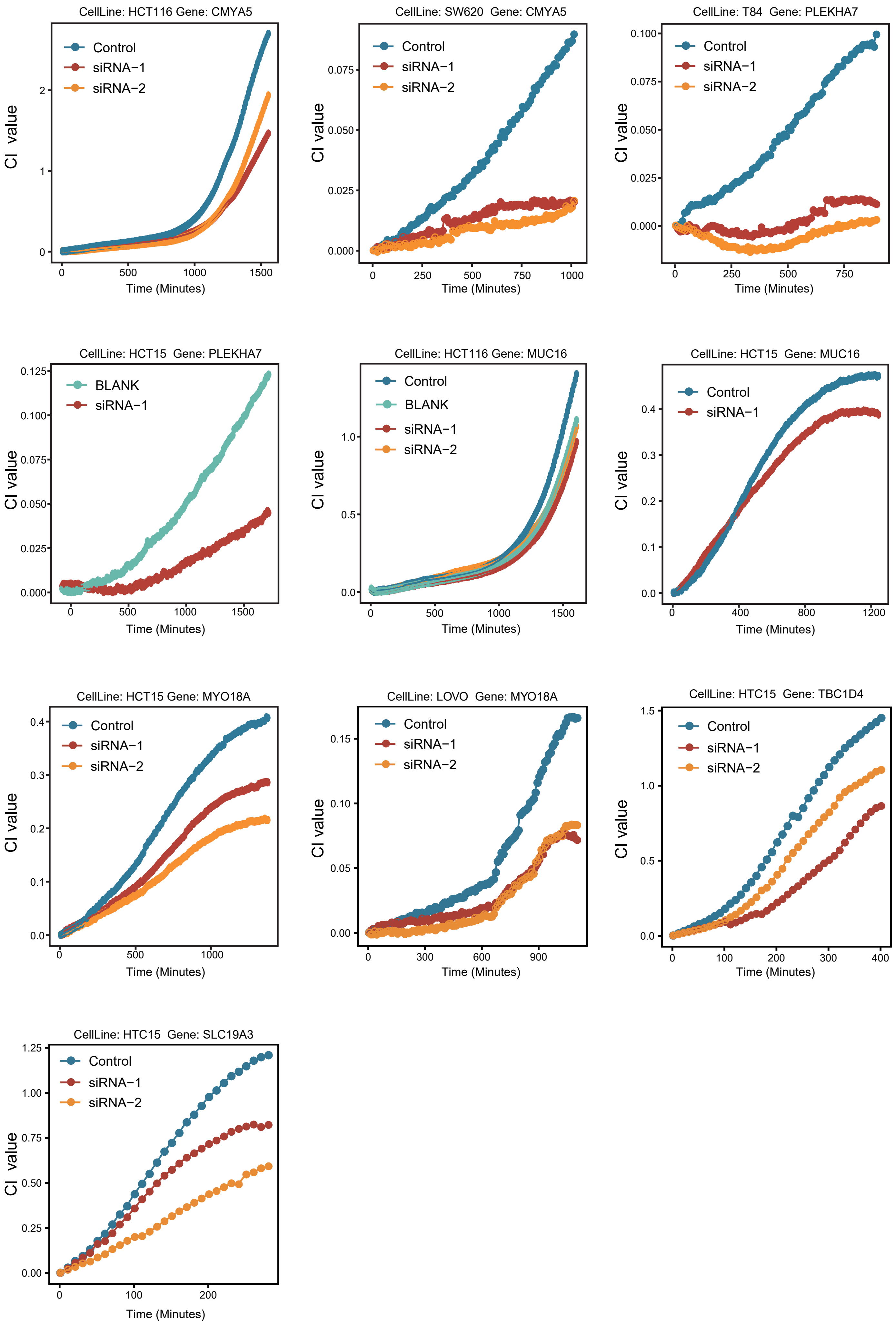

CellLine: HCT116 Gene: CMYA5
Control
siRNA−1
siRNA−2
CI value
Time (Minutes)
CellLine: SW620 Gene: CMYA5
Control
siRNA−1
siRNA−2
CI value
Time (Minutes)
CellLine: T84 Gene: PLEKHA7
Control
siRNA−1
siRNA−2
CI value
Time (Minutes)
CellLine: HCT15 Gene: PLEKHA7
BLANK
siRNA−1
CI value
Time (Minutes)
CellLine: HCT116 Gene: MUC16
Control
BLANK
siRNA−1
siRNA−2
CI value
Time (Minutes)
CellLine: HCT15 Gene: MUC16
Control
siRNA−1
CI value
Time (Minutes)
CellLine: HCT15 Gene: MYO18A
Control
siRNA−1
siRNA−2
CI value
Time (Minutes)
CellLine: LOVO Gene: MYO18A
Control
siRNA−1
siRNA−2
CI value
Time (Minutes)
CellLine: HTC15 Gene: TBC1D4
Control
siRNA−1
siRNA−2
CI value
Time (Minutes)
CellLine: HTC15 Gene: SLC19A3
Control
siRNA−1
siRNA−2
CI value
Time (Minutes)

Fig S5: Real-time impedance traces for CRC cell lines transfected with siRNAs specific to metastatic tumour clone-specific mutated genes. Cell lines and gene names are labelled on the top of each panel. The control siRNA and target gene siRNAs are colour-coded based on that described in the legends.
